# Treatment Considerations for Compulsive Exercise in High-Performance Athletes with an Eating Disorder

**DOI:** 10.1186/s40798-022-00425-y

**Published:** 2022-03-03

**Authors:** Jordan A. Martenstyn, Nikki A. Jeacocke, Jana Pittman, Stephen Touyz, Sarah Maguire

**Affiliations:** 1 Clinical Psychology Unit, School of Psychology, The University of Sydney, Sydney, NSW Australia; 2 InsideOut Institute for Eating Disorders, Charles Perkins Centre, The University of Sydney, Sydney, NSW Australia; 3 Australian Institute of Sport, Canberra, ACT Australia; 4 School of Medicine, Blacktown Hospital, Western Sydney University, Sydney, NSW Australia; 5 Sydney Local Health District, NSW Health, St Leonards, Sydney, NSW Australia

**Keywords:** Compulsive exercise, Exercise addiction, Exercise abstinence, High-performance sport, Elite athlete, Eating disorder, Anorexia nervosa, Bulimia nervosa, CBT-E

## Abstract

Compulsive exercise is linked with poorer treatment outcomes in people with eating disorder (EDs). High-performance athletes represent a growing and complex subcomponent of the broader ED population, and emergent evidence indicates that different conceptualisations of compulsive exercise are needed in this population. Existing randomised controlled trials in ED populations have demonstrated small treatment effects on compulsive exercise compared with control groups; however, athletes were sparsely sampled across these studies. Thus, the extent to which current treatments for compulsive exercise in EDs are also effective in high-performance athletes is unknown. For this opinion paper, we sought representation from high-performance sports leadership, someone with lived experience of both an ED and high-performance athletics, and ED clinical experts. We discuss the utility of recommending exercise abstinence in ED treatment with athletes, as well as a number of other treatment strategies with some evidence in other contexts for further consideration and research in this population. These include using mindfulness-based interventions as an adjunct to cognitive-behavioural therapies, using wearable technologies and self-reported fatigue to inform training decisions, and incorporating greater exercise variation into training programs. We also offer practical considerations for clinicians seeking to apply foundational elements of cognitive-behavioural interventions (e.g., exposure and response prevention, cognitive restructuring, behavioural experiments) into an ED treatment program for a high-performance athlete. Future research is needed to examine characteristics of pathological compulsive exercise in athletes and whether available treatments are both feasible and effective in the treatment of compulsive exercise in athletes with an ED.

## Key Points

1. There is limited research on how to manage and treat compulsive exercise in high-performance athletes with an eating disorder.
2. While sport and exercise abstinence is often needed in people with a severe eating disorder with significant medical risk, there are several drawbacks that should be considered when recommending sport and exercise abstinence in a high-performance athlete with an eating disorder of mild or moderate severity.
3. Several strategies offer promise in treating compulsive exercise in high-performance athletes and warrant further research, including incorporating elements from mindfulness-based interventions, using wearable technologies to inform training decisions, and including greater exercise variation into a training program.

## Introduction

Unhealthy exercise has long been considered a core feature of anorexia nervosa (AN) and bulimia nervosa. Earlier researchers used the term excessive exercise to describe this pattern of exercise behaviour often observed in people with eating disorders (EDs), purporting specific quantitative limits to demarcate *excessive* from *non-excessive* exercise [1]. The current perspective, however, is that unhealthy exercise should be characterised according to its qualitative (rather than solely its quantitative) dimensions, since what is considered ‘excessive’ depends on a number of individual factors, including age, health status, fitness abilities [2, 3], and potential classification as a high-performance athlete. Consistent with existing literature, we define a high-performance athlete as someone who competes in sporting competitions at a national or international level, encompassing both junior and senior athletes, para- and able-bodied athletes, and individual and team sport athletes [4].

At present, compulsive exercise is the most commonly used term amongst ED professionals to describe unhealthy exercise [5]. Dittmer et al. [2] proposed a transdiagnostic definition of compulsive exercise in EDs based on the driven and rigid nature of exercise behaviour, significant interference in normal life, and degree of self-insight (Table 1). Compulsive exercise is associated with a number of deleterious outcomes in people with EDs [6] and the treatment of compulsive exercise has garnered the attention of two recent reviews [7, 8]. In the Martenstyn et al. [8] meta-analysis, we found that almost all existing interventions for compulsive exercise included multiple treatment components, including psychoeducation, structured exercise, and/or psychotherapy, with only three studies evaluating single-component interventions [8]. Across the four available randomised controlled trials (RCTs) evaluating treatments for compulsive exercise, small reductions (Cohen’s *d* = − 0.23) were found across a range of ED diagnoses when compared with control groups [8].

**Table 1:** Definition of compulsive exercise applied to high-performance athletes

Criterion A (obligatory)	Translation to high-performance athletes
A1: Excessive exercise that the patient feels driven to perform in response to an obsession or according to rules that must be applied rigidly	High-performance athletes are generally highly motivated and driven to exercise to improve their sporting performance, which some may conceptualise as an “obsession”. High-performance athletes also frequently adhere to strict training programs, which could be interpreted as “rules that must be applied rigidly”
A2: The exercise is aimed at preventing some dreaded consequence or at preventing or reducing distress, often based on distorted beliefs about exercise	Many high-performance athletes exercise to increase performance and fitness, but also due to a fear of failure (“some dreaded consequence”) in sports competition [65]. Exercise may also be used by some competitive athletes to regulate affect, particularly the avoidance of negative affect [23]

Criterion B (obligatory)	Applicability to elite athletes
The compulsive exercise is time-consuming (takes more than 1 h a day)	The training program of a high-performance athlete is often very time-consuming and requires on average multiple hours of training per day during the week
[The compulsive exercise] significantly interferes with the person’s daily routine, occupational functioning or social relationships	Being a high-performance athlete requires life activities to be structured around training and competition, rather than training and competition to be structured around life activities
[The compulsive exercise] is continued despite medical injury, illness, or lack of enjoyment	High-performance athletes often train through illness and injury. Lack of exercise enjoyment may occur during extremely intense sessions, extended competition periods, or episodes of burnout [66]

Criterion C (optional)	Applicability to elite athletes
At some point during the course of the disorder the patient has recognized that the compulsive exercising is excessive or unreasonable	Although some high-performance athletes show insight that their training is unsustainable (which may coincide with retirement), there is little empirical research on this topic [36]

Adapted from Dittmer et al. [2] under Creative Commons license: (https://s100.copyright.com/AppDispatchServlet?title=Compulsive%20exercise%20in%20eating%20disorders%3A%20proposal%20for%20a%20definition%20and%20a%20clinical%20assessment&author=Nina%20Dittmer%20et%20al&contentID=10.1186%2Fs40337-018-0219-x&copyright=The%20Author%28s%29.&publication=2050-2974&publicationDate=2018-11-28&publisherName=SpringerNature&orderBeanReset=true&oa=CC%20BY%20%2B%20CC0)

Rates of EDs and compulsive exercise differ as a function of gender and sport domain. In the general population, women are almost four times as likely as men to develop an ED over their lifetime [9] and are more susceptible to ED behaviours within a given sport [10, 11]. Rates of EDs and disordered eating are also elevated in high-performance athletes compared with the general population, particularly in athletes competing in aesthetically judged (e.g., gymnastics, bodybuilding), gravitational (e.g., running), and weight class sports (e.g., martial arts) [11–14]. While levels of compulsive exercise are higher in men than women in the general population, this gender difference appears less pronounced in high-performance sport [15, 16]. Overall, levels of compulsive exercise are higher in high-performance athletes compared with both recreational and non-athletes, and appear greatest in athletes involved in endurance and ball sports [17–19]. Despite evidence that high-performance athletes are at greater risk of an ED and compulsive exercise, high-performance athletes have largely been excluded from past studies on the treatment of compulsive exercise and/or EDs [20, 21]. Where research did sample current or former athletes, athletic status was not an explicit consideration of the treatment intervention [22].

This methodological decision to exclude athletes is understandable as there are several unique challenges associated with sampling athletes with an ED that are not present with non-athletic populations. These include: (a) increased participant heterogeneity if not all included participants are athletes; (b) treatment adherence could be poor if treatment sessions are scheduled during the competition season, particularly if travel is involved; (c) treatment will need to be adapted to account for the athlete’s increased energy expenditure; and (d) treatment will likely require cooperation with external parties (e.g., sporting clubs and coaches) who may have conflicting goals to the clinician. Due to these practical considerations, little is known about how to adapt current treatment programs for high-performance athletes with an ED and/or compulsive exercise. This article discusses a number of treatment considerations related to both the assessment and treatment of compulsive exercise in athletes with an ED.

## Translation of the Compulsive Exercise Construct to Competitive Athletes

One important milestone for ED treatment is the successful reduction of compulsive exercise, if present. However, some of the criteria used to define compulsive exercise in EDs [2] are hallmark behaviours of competitive athletes, for example, exercising to control weight or regulate affect [23]. It could therefore be argued that compulsive exercise – as currently defined in the literature – represents a series of attitudes and behaviours that are critical to becoming a successful athlete, making it difficult to generalise available research from non-athletes to athletes. Indeed, different factor structures have been reported for the Compulsive Exercise Test (CET) between athletes and non-athletes. In the original validation of the CET which recruited from the general population, the authors found evidence for a five-factor structure, including: (a) avoidance and rule-driven behaviour; (b) weight control exercise; (c) mood improvement; (d) lack of exercise enjoyment; and (e) exercise rigidity [24]. Further validations of the CET in competitive athletes indicated a three-factor structure, consisting of: (a) avoidance of negative affect; (b) weight control exercise; and (c) mood improvement [23]. Overall, there appear to be differences in how compulsive exercise should be conceptualised between athletes and non-athletes. Consistent with this notion, research suggests that increased rates of compulsive exercise in competitive athletes compared with the general population [18] could be an artefact of increased passion and dedication to their sport [25, 26]. Thus, alternative or modified measures of compulsive exercise are needed in athletic populations to distinguish potentially pathological and harmful behaviours from those necessary for sporting excellence.

It is insufficient to assess pathological compulsive exercise in athletes based solely on the quantity of sport-specific exercise behaviours, as almost all high-performance athletes would appear to meet existing compulsive exercise criteria from this perspective. With representation from high-performance sports leadership (Australian Institute of Sport), lived experience of both an ED and high-performance sport, and ED clinical experts, we have identified two potential behavioural indicators of clinically significant compulsive exercise specific to high-performance athletes. These include: (a) whether the athlete has flexibility to reduce their exercise in certain instances, including between major competitions or seasons, during injury and/or illness, or during a prescribed reduction in training load (e.g., a taper), without experiencing substantial distress and guilt; and (b) whether the athlete performs additional exercise outside of scheduled training sessions, either to manipulate weight and/or body composition, or for affect regulation purposes. Evidence suggests that understanding an athlete’s degree of compulsion to exercise can inform appraisal of their ED severity [27]. Overall, it is unclear from the existing literature whether compulsive exercise offers a useful measure of psychological dysfunction in athletes with an ED. Greater understanding of the dimensions of compulsive exercise that translate from non-athletes to athletes represents an important precursor to successful adaptation of existing ED treatments and assessments.

## Treatment Considerations for Clinicians

Given the maintaining role of compulsive exercise in ED symptomology, clinicians face a dilemma as to whether an ED can be successfully treated in an athlete who continues to ‘compulsively exercise’ to meet the demands of their sport. Our clinical experience highlights the difficulties associated with treating an ED without being able to make relevant changes to exercise behaviour, either through partial or complete abstinence, or recommending alternative forms of exercise. However, there is a dearth of research exploring the treatment factors that affect ED prognosis in high-performance athletes.

One treatment approach often used in ED treatment regardless of athletic status has been to disregard the client’s sporting and exercise goals, and encourage complete abstinence from sport and exercise until significant reductions in ED symptomology have been achieved [28, 29]. Once the ED is perceived to be manageable, participation in sport and exercise can be reintroduced in a graded fashion. Supporters of this perspective might argue that EDs, particularly anorexia nervosa, have among the highest mortality rates of any psychiatric illness [30], which necessitates complete abstinence from sport regardless of the short-term costs. While drastic, this treatment approach is consistent with existing guidelines proposing exercise abstinence in severe ED cases where there is significant medical risk [29, 31].

However, the decision of whether to incorporate sport and exercise abstinence in people with an ED of mild or moderate severity is more nuanced. Several reviews indicate that inclusion of structured exercise performed under supervision does not interfere with treatment goals of weight restoration in underweight patients with an ED [32–34]. As such, current guidelines advocate for the gradual inclusion of structured, supervised, and non-aerobic (if severely underweight) forms of exercise into an ED treatment program, provided that medical clearance has been granted [29, 31]. However, clinicians must also consider the extent to which the high-performance sporting environment (e.g., need for strict weight control, external appearance pressures, exposure to other athletes with disordered eating behaviours) perpetuates the ED and whether participation in that cultural environment is compatible with ED recovery [27, 35].

Considering this evidence base, there are a number of important considerations when recommending complete exercise abstinence in athletes with an ED of mild or moderate severity. Firstly, many athletes derive a large portion of their income, self-esteem, and sense of identity from their sporting performances, and could suffer notable financial (e.g., loss of income) and/or psychiatric distress if complete abstinence from sport was prescribed without sufficient evidence to support its efficacy [36, 37]. ED symptoms may also temporarily worsen or treatment compliance can decrease when exercise behaviour is involuntarily restricted, either from injury or abstinence [38, 39]. Secondly, athletes might be reluctant to pursue treatment for an ED if there is a perceived risk that a clinician will advocate for temporary retirement from sport or break confidentiality by sharing such information with support persons [40]. Thirdly, it generally takes 20–40 sessions of psychotherapy for clients to experience notable reductions in ED symptoms and many clients take up to a decade to achieve recovery from an ED [41], making it difficult to forecast specific durations for abstinence. Finally, several RCTs including a structured exercise component into multi-modal treatment have demonstrated significant reductions in compulsive exercise in people with EDs [20, 21, 42], suggesting that complete abstinence from exercise is not essential to treat compulsive exercise. Overall, the decision of whether to recommend complete abstinence from sport should be made carefully in the context of a number of factors, including ED severity, medical stability, client motivation, and the client’s dependence on their sport, both financially and psychologically [28, 29]. Certainly, when severe medical complications outweigh psychological, financial or other losses from exercise abstinence, treatment of the ED should not be withheld for any professional purpose, including sport [28, 29]. In this article, we put forth a number of other treatment strategies—in addition to complete abstinence—aimed at reducing compulsive exercise in athletes with an ED for further consideration and research.

### Education

Psychoeducation is an essential component of ED treatment and general discussion topics have been described in detail in seminal ED treatment manuals [43, 44]. For athletes, fundamental principles of sports medicine (e.g., energy availability) and body diversity may also be discussed. While there is no research comparing the efficacy of ED-specific psychoeducation material delivered individually with a client versus with support persons such as coaches and dietitians, clinical experience suggests that shared understanding between the clinician, athlete, and support staff can be enhanced when psychoeducation is delivered with support persons present. Clinicians are also encouraged to allocate sufficient time to explain the rationale for ED treatment to both the athlete and support staff to reduce the risk of support persons inadvertently reinforcing ED and/or compulsive exercise.

The link between compulsive exercise, low energy availability (LEA), and relative energy deficiency in sport (RED-S) offers a useful education topic for athletes and performance support staff. Energy availability is the amount of energy remaining to support the body’s health and functioning once energy expenditure from exercise is subtracted from dietary energy intake [45]. LEA results in inadequate energy to cover the body’s daily needs outside of exercise, and arises from insufficient dietary energy intake, excessive exercise energy expenditure, or a combination of both [45]. RED-S is a syndrome that arises from LEA and is associated with impaired metabolic rate, menstrual function, immune health, and cardiovascular health, amongst other things [27]. Our clinical experience suggests that discussing the importance of adequate energy availability to reduce the risk of RED-S and the potential negative impact it can have on performance often resonates with athletes and support staff.

The main principle of body diversity is that different athletes perform best at different shapes and sizes. While there are undeniable differences in body shape between athletes from disparate sports (i.e., a 100 m sprinter compared with  a marathon runner), there is variation in the exact weight and shape that each athlete *within* a given sport associates with optimal performance. As such, staff involved in the planning of an athlete’s diet are encouraged to base food choices around what maximises performance for the athlete as an individual, rather than in an attempt to meet a pre-conceived (and potentially incorrect) assumption about the anthropometric proportions of a successful athlete.

### Mindfulness to Enhance Body Awareness

Two systematic reviews indicate that mindfulness-based interventions can be effective in non-athletic populations at reducing ED behaviours, overvaluation of weight and shape, and improving emotion regulation [46, 47]. As such, clinicians may find mindfulness a useful adjunct in a multi-component ED intervention to help athletes become more attuned to their internal physical sensations (e.g., fatigue and muscle soreness), which often precedes future development of injuries or overtraining syndrome [48]. We suggest that mindfulness could be performed initially in-session with the guidance of the clinician, and later progress to being performed alone by the athlete or with the guidance of a coach before/after sport activities. Further empirical research is needed to determine whether mindfulness has adjunctive benefits with enhanced cognitive behavioural therapy (CBT-E) for reducing compulsive exercise and ED symptomology, both in non-athletes and athletes with an ED.

### Wearable Technologies to Assess Recovery

Wearable technologies have received considerable public and scientific attention in the past half-decade as non-invasive tools to gather biometric data in athletes and the general population. A growing body of literature supports the efficacy of these wearable technologies in assessing physiological variables such as heart rate variability (HRV) and sleep architecture [49–52]. Many high-performance sporting teams integrate data from wearable technologies into an athlete monitoring system to assess an athlete’s recovery. These technologies offer promising tools to inform decision-making in a sporting context, such as when to schedule a hard training session or rest an athlete. However, one potential risk that should be closely monitored by clinicians working with high-performance athletes with an ED is that wearable technologies may drive some athletes to compulsively increase exercise output to meet specific targets (e.g., step count, calories burned), and override internal markers of recovery [53].

### Matching Training Intensity to Recovery

One focus of ED treatment for athletes could be to work with them and/or their support team to learn to match the intensity of training sessions to their recovery, assessed using wearable technologies (objectively), and self-reported fatigue and muscle soreness (subjectively). For example, where medically and psychiatrically appropriate, if an athlete feels energetic, reports minimal muscle soreness, and wearable technologies indicate sufficient recovery (e.g., low resting heart rate and/or high HRV), a higher-intensity and/or longer duration training session could be scheduled [54]. In contrast, if the athlete feels exhausted, reports substantial muscle pain, and wearable technologies suggest low preparedness to exercise, a rest day or active recovery day could be scheduled. When there are discrepancies between subjective and objective indicators of fatigue, subjective measures could be prioritised and wearable technologies considered a helpful (but imperfect) supporting tool. Such a flexible approach to organising training may not lend itself to traditional experimental research designs, but a case report methodology assessing exercise enjoyment and compulsive exercise could provide an initial empirical analysis of this clinical approach.

### Exposure and Response Prevention

Exposure and response prevention (ERP) is one of the core treatment strategies for compulsive behaviours, such as obsessive–compulsive disorder [55]. It has been incorporated into several multi-modal interventions addressing compulsive exercise in people with EDs, but not specifically in athlete samples [42, 56]. ERP can be administered in session with athletes by: (a) exposing them to cues that increase their urge to exercise (e.g., footage of a competitor winning a major competition); and then (b) asking them to remain still until their urge to exercise drops to baseline levels. We have observed clinically that assisting athletes to find pleasurable distraction activities, such as reading, meditating, or knitting, has been helpful in managing their urges to exercise when alone or distressed.

### Cognitive Restructuring

Central to a cognitive behavioural therapy (CBT) intervention for EDs is a discussion of beliefs around the role of exercise in weight management and as a means for reward or punishment [43, 44]. In traditional CBT-E, clients are asked to regularly self-monitor their food intake, exercise behaviour, and associated thoughts and emotions [43, 44]. It is important that clinicians use these monitoring forms to assess the extent to which exercise motivations are related to appearance intolerance, performance reasons, functional goals (e.g., to feel strong), and/or emotion regulation. If an athlete endorses high appearance-based motivations, the ED clinician could utilise regular cognitive restructuring techniques (e.g., a pros and cons list) to help the athlete become aware of the downsides of exercising primarily for appearance reasons and to consider other more functional- and performance-based benefits of exercise. Other athletes might endorse a rigid belief that food intake is dependent on athletic performance, such that a poor or missed training session should be ‘punished’ with reduced or no food intake. Such a belief could be addressed using a behavioural experiment (Table 2). While cognitive restructuring is a core feature of CBT-E, anecdotal experience and previous research suggest that ED clinicians are often hesitant or unaware how to treat compulsive exercise, which could represent a barrier to delivering effective psychotherapy [28].

**Table 2:** Potential behavioural experiments for athletes with an eating disorder

Description of experimental conditions	Variables assessed
1a: Adopt a flexible training schedule for two weeks where the intensity of training sessions is matched to objective/subjective indicators of recovery 1b: Continue with planned training schedule regardless of objective/subjective indicators of recovery	Satisfaction with training Exercise enjoyment Self-reported fatigue Coach rating of training performance
2a: Incorporate exercise variety into training program for one week 2b: Incorporate no exercise variety into training program for one week	Satisfaction with training Exercise enjoyment Self-reported fatigue Heart rate variability Coach rating of training performance
3a: Increase food intake after a poor or missed training session 3b: Reduce food intake after a poor or missed training session	Self/coach rating of performance at subsequent training session Exercise enjoyment Self-reported fatigue Heart rate variability

### Designing Effective Behavioural Experiments

The testing of distorted beliefs and assumptions about food and exercise with behavioural experiments is one of the foundational elements of CBT-E [43, 44]. However, it is difficult to design an effective behavioural experiment to test cognitions about exercise in high-performance athletes who are unable to make significant changes to their exercise routine. In Table 2, we suggest a number of practical behavioural experiments that can be conducted with athletes presenting for ED treatment at any stage throughout the year. No research has explored the feasibility of conducting behavioural experiments in athletes with an ED between competition periods, but we suggest that behavioural experiments that involve substantial modifications to exercise behaviour could be scheduled for non-competition periods when training demands are lower.

### Increasing Exercise Variety

Treatment interventions that produced significant reductions in compulsive exercise in non-athletes with EDs included appropriate variation within their exercise programs [20, 21, 42]. Alternating the choice of exercises between workouts is linked with greater intrinsic exercise motivation and exercise adherence in the general population [57–59]. It can also reduce exercise-related boredom, which is associated with reduced performance in high-performance athletes across both individual and team sports [60]. Incorporating variation into an exercise program can be accomplished via: (a) alternating between different modes of cardiovascular exercise used to increase aerobic fitness, for example, jogging, swimming, cycling, and rowing [59]; and/or (b) adding novel exercises to active recovery days, such as pilates, hiking, or rock climbing, to promote engagement of different muscles and kinesthetic experiences. We advocate that clinicians discuss collaboratively with their client and associated support staff how exercise variation could be incorporated into their training program.

### Developing Alternative Emotion Regulation Strategies

Regulating emotions without the use of food or exercise is a challenge for many people with EDs [61, 62]. Clinicians working with athletes with an ED are encouraged to conduct a functional analysis of exercise, including discussion of recent instances when the client felt compelled to exercise [2]. Athletes may exercise individually outside of group training sessions and/or use exercise in planned training sessions as an outlet to manage arousal and distress. Enquiries about whether the athlete exercises outside of planned training sessions, and their affect pre- and post-training, can form a useful part of assessment. If the client demonstrates an overreliance on exercise or food to manage emotions, they could be guided to consider alternative evidence-based emotion regulation strategies, such as acceptance, mindfulness, distraction, and reappraisal of the emotional stimulus [63, 64].

## Conclusion

High-performance athletes represent a growing and complex subcomponent of the broader ED population and it is unclear the extent to which current conceptualisations of, and treatments for, compulsive exercise translate to sporting populations. Clinicians working with high-performance athletes with EDs face a number of unique challenges and some have adopted the perspective that abstinence from sport is a requirement for successful ED treatment. In this article, we discuss the limitations of this treatment approach for people with an ED of mild or moderate severity, and the utility of a range of other clinical tools to address problematic exercise behaviour in athletes. Further qualitative and quantitative research with athletes, coaches, and clinicians is needed to understand whether existing treatment strategies from CBT-E and those proposed in this article are both feasible and effective for reducing compulsive exercise in high-performance athletes with an ED.

## Data Availability

Not applicable.
